# Case of Acute Tetanus Following a Wound in the Foot

**Published:** 1878-01

**Authors:** 


### Selections.

Case of Acute Tetanus following a Wound in the Foot.— The following case of tetanus is, I think interesting:

Geo. Smith, aged 10, a fisherman's son of Sambro, on August 2d, whilst barefoot, accidentally ran against a scythe, causing a wound of left instep implicating the extensor tendons, also one nearly severing the little toe of the same foot.

He was brought to Halifax and the wound dressed; he then returned. On August 9th I saw him for the first time; wound of instep granulating healthily, a small piece of bone protruding from wound of little toe, which was removed. Sweating profusely, trismus and opisthotonos well marked, with frequent spasms, difficulty of diglutition and respiration, pulse 144, temp. 103 degrees, pain through body, risus sardonicus marked. Gave gr. xvi. hydrate chloral immediately and gr. viii to be given every hour with mustard to whole length of spine, wound dressed with carbolic acid and oil (1-20). Remained all night and administered the chloral myself, with as much milk as he could suck through the teeth and on account of the great difficulty in swallowing, it required much patience to give either. The boy soon slept and was awakened during the night to take chloral and milk. Spasms were much less frequent after the chloral.

Aug. 11th. Worse again, boy refusing chloral, also milk, temp. 101¾ degrees, pulse 130 and weak, sweating profusely, frequent spasms, no relief of trismus or opisthotonos,—remained three hours giving gr. viii, chloral every hour. Gave also during this time half pint of milk. Left him much relieved.

Aug. 14th. Not any worse, pulse 116, temp. 99 degrees, but few spasms, trismus very slightly relieved, spine still stiff but limbs can be bent up on abdomen if care is used. Grains viii chloral every hour, takes but very little milk, bowels relieved by enema, passed quantities of flatus.

Aug. 17th. Was doing well since the last visit, but the boy refusing chloral, the spasms returned to some extent. Gave gr. xvi of chloral, with marked relief and ordered gr. viii of chloral every two hours with as much milk as possible. Boy very weak.

Aug. 20th. Boy better, pulse 98, temp. 99 degrees, spasms few and slight, trismus somewhat relaxed, bowels moved by injection, still fights against medicine and nourishment.

Aug. 23d. Decidedly improving, pulse 88, temp. 98 degrees, jaws still more relaxed, scarcely any spasms except when moved and even then slight, wounds nearly healed. Gave chloral gr. vii every four hours which was continued for a week longer and then given only at night. From this time continued to improve, and on September 7th the boy's father reports that his jaws are looser than ever.

This case is interesting in that it is a recovery from a very acute case, which is rare, that no drug except chloral was administered, no stimulants, and nothing but milk and very little of that for a fortnight.—*Canada Medical and Surgical Journal.*

